# Need for speed: bacterial effector XopJ2 is associated with increased dispersal velocity of *Xanthomonas perforans*

**DOI:** 10.1111/1462-2920.15541

**Published:** 2021-06-08

**Authors:** Anuj Sharma, Sujan Timilsina, Peter Abrahamian, Gerald V. Minsavage, James Colee, Peter S. Ojiambo, Erica M. Goss, Gary E. Vallad, Jeffrey B. Jones

**Affiliations:** ^1^ Department of Plant Pathology University of Florida Gainesville Florida USA; ^2^ Gulf Coast Research and Education Center University of Florida Wimauma Florida USA; ^3^ Statistics Consulting Unit, Institute of Food and Agricultural Sciences University of Florida Gainesville Florida USA; ^4^ Department of Entomology and Plant Pathology North Carolina State University Raleigh North Carolina USA; ^5^ Emerging Pathogens Institute University of Florida Gainesville Florida USA

## Abstract

Bacterial spot caused by *Xanthomonas perforans* (*Xp*) is an economically important disease in tomato. Previous studies have shown that the recently isolated *Xp* strains have acquired and retained the effector gene, *xopJ2*, which has been reported to increase fitness of the pathogen in the field. To elucidate the fitness benefit of *xopJ2*, we quantified the effect of *xopJ2* on the dispersal and evolution of *Xp* populations on tomato. We compared movement of two wild‐type *Xp* strains expressing *xopJ2* to their respective *xopJ2* mutants when co‐inoculated in the field. We developed a binary logistic model to predict the presence of *Xp* over spatial and temporal dimensions with or without *xopJ2*. Based on the model, wild‐type bacteria were dispersed approximately three times faster than the *xopJ2* mutants. In a simulation experiment, the selective advantage due to increased dispersal velocity led to an increase in the frequency of *xopJ2* gene in the *Xp* population and its apparent fixation within 10 to 12 cropping seasons of the tomato crop. Our results show that the presence of a single gene can affect the dispersal of a bacterial pathogen and significantly alter its population dynamics.

## Introduction

Plant pathogenic bacteria are a major threat to modern agriculture. The repertoire of pathogenicity factors utilized by bacteria for establishing disease, such as secretion systems, have been well‐elucidated in plant pathology (Dangl and Jones, 2001; Büttner and He, 2009; White *et al*., 2009; Gordon *et al*., 2015; Levy *et al*., 2018). The dynamic nature of bacterial populations has been clearly illustrated by several large‐scale epidemiological and phylogenetic studies; however, the genetic bases underlying these evolutionary changes remain poorly understood (Mhedbi‐Hajri *et al*., 2013; Newberry *et al*., 2019; Pruvost *et al*., 2019). The genetic elements that are under selection can dictate the direction of bacterial evolution. Understanding how a gene under selection influences the bacterial population can help to decipher its role in pathogen fitness. Towards this goal, the effects of a single gene can be studied at a small spatial scale, i.e., microenvironment, and the results can be carefully extrapolated over consecutive cropping periods in a closed system to evaluate its impact on the overall population.

Bacterial spot of tomato (BST) is an economically devastating disease caused by several *Xanthomonas* spp., including *Xanthomonas perforans* (*Xp*) and *X*. *euvesicatoria* (*Xe*) (Jones *et al*., 2004). High yield losses can occur if the environmental conditions are conducive for pathogen dispersal and disease development (Pohronezny and Volin, 1983). The pathogens surviving in crop residue and plant debris has been reported as a primary source of inoculum for transmission of BST to subsequent generations of tomato, although movement from nursery seedlings has been reported and seed‐transmission has also been speculated (Jones *et al*., 1986; Abrahamian *et al*., 2019). Bacterial spot of tomato has been extensively studied in Florida with strain collections showing that the population of BST‐associated xanthomonads underwent a dramatic shift in genetic structure in the two decades starting in early 90s. Until the early 1990s, *Xe* (also referred to as tomato race 1 (T1)) was the sole *Xanthomonas* sp. reported to cause BST in Florida (Jones *et al*., 1998). A new amylolytic and pectolytic strain of BST pathogen was identified in 1991 and was designated race T3 and later as a new species, *Xp* (Jones *et al*., 1995, 2004). Since then, *Xp* has completely replaced *Xe* in Florida, most likely because of its antagonistic bacteriocin activity against *Xe* (Hert *et al*., 2005; Timilsina *et al*., 2016). Even within *Xp*, strains showed directional evolution over time. In 1998, a new race of *Xp* was isolated which, unlike the strains of *Xp* isolated before, lacked a functional AvrXv3 effector, and was designated race T4 (Horvath *et al*., 2012). By 2012, all BST‐causing strains isolated in Florida were *Xp* T4 strains (Timilsina *et al*., 2016). Furthermore, recently collected field strains of *Xp* have been classified into two phylogenomic groups: 1 and 2, where group 1 strains are close to the reference strain 91‐118 and group 2 strains share more sequence similarity with *Xe* (Schwartz *et al*., 2015). A third group was also recently described from *Xp* strain collection (Abrahamian *et al*., 2019; Timilsina *et al*., 2019).

The appearance of exotic bacterial strains may result from modifications in the effector profile of a bacterial species, due to gene loss (such as mutation or deletion), or introduction of novel effectors by horizontal gene transfer (HGT) (Fraser‐Liggett, 2005). Of special importance are type‐III secreted effectors (T3SEs), small proteins secreted by the type III secretion system directly into host cells, where they alter transcription, hijack cell signalling, and suppress host defences (Büttner, 2016). In studies of changes in *Xp* effector profiles, several effectors were identified to be retained in the overall *Xp* population (Timilsina *et al*., 2016). One of the T3SEs that really stood out was *Xanthomonas* outer protein J2 (XopJ2, also referred to as AvrBsT), a plasmid‐borne *Xanthomonas* T3SE. It belongs to the YopJ/AvrRxv family of effectors, which have been reported in both animal‐ and plant‐pathogenic bacteria to possess cysteine protease and acetyltransferase activity (Cheong *et al*., 2014; Ma and Ma, 2016). It was initially characterized in *Xe* strain 75‐3 as an avirulence gene that induced a hypersensitive response (HR) in pepper but not in tomato (Minsavage *et al*., 1990). In *Arabidopsis*, *xopJ2* has been reported to interfere in microtubule formation through acetylation of tubulin‐binding protein ACIP1 (Cheong *et al*., 2014). In pepper, *xopJ2* can suppress effector‐triggered immunity (ETI) due to recognition of AvrBs1 (Szczesny *et al*., 2010). Field surveys from tomato production fields in Florida showed changes in prevalence of *xopJ2* gene in *Xp* population over time (Timilsina *et al*., 2016). The original T3 strain of *Xp* isolated in 1991 did not possess the *xopJ2* gene. *xopJ2* was first found in *Xp* in a 1998 isolate, which was also the first T4 strain to be isolated (Horvath *et al*., 2012). The sequence of *Xp xopJ2* was identical to that from *Xanthomonas vesicatoria*, another BST causing *Xanthomonas* spp., indicating that *Xp* acquired *xopJ2* through HGT (Timilsina *et al*., 2016). Following the first detection of *xopJ2* in *Xp* in 1998, the frequency of the gene in the population has risen dramatically, with almost 75% and 100% of bacterial strains isolated in 2006 and 2012, respectively, containing *xopJ2* (Timilsina *et al*., 2016).

Strains of *Xp* have acquired and retained the *xopJ2* gene, indicating it may confer a selective advantage. Fitness is the relative contribution of individuals to the next generation, and in case of bacterial pathogens, fitness can also be assessed by the change in the distribution of a gene or genetic element over time in the host population. To determine if *xopJ2* confers a fitness advantage, Abrahamian *et al*. (2018) compared the growth pattern of wild‐type (WT) *Xp* strains with functional *xopJ2* gene to their respective near‐isogenic mutants with non‐functional *xopJ2*. The presence of the *xopJ2* gene did not significantly affect the *in‐planta* population level of *Xp*, however, WT *Xp* was recovered from longer distances from the point of inoculation than the respective mutant in field experiments.

Dispersal and distribution of bacteria can be studied by developing spatiotemporal dispersal models, which predict the probability of pathogen incidence over time and distance. Essentially, these are regression models developed from disease observations made in the field/nursery. Logistic models, which transform observed probability to log‐odds before model fitting, are commonly used to describe dispersal based on binary (i.e., presence/absence) data such as pathogen incidence. Unfortunately, simple linear models are often not very useful for biological/ecological data as they make several assumptions (such as normality) that are not valid for such data (Bolker *et al*., 2009). More advanced regression models such as generalized linear mixed models (GLMM) are suitable for biological data which are often not normally distributed affected by many factors, some of which may not be important towards the objectives of the study (Bolker *et al*., 2009) (see Supporting Information S1 and S2 for further discussion). Modelling starts with a full model, which contains many potentially important predictors and their interactions. Then, predictors that do not significantly contribute to the model are dropped serially to develop the final model (see Supporting Information S3 for significance tests). Candidate models need to be tested for their goodness‐of‐fit and predictive power before inferences can be made based on their predictions (Funk *et al*., 2019). This is often done by calculation of goodness‐of‐fit parameters and binary classification metrics, residual analysis, generation of receiver operating characteristics (ROC) curve, and cross validation (see Supporting Information S4–S7).

Abrahamian *et al*. (2018) demonstrated the possible fitness role of *xopJ2* in *Xp* dispersal, but did not study the magnitude of relative fitness provided by *xopJ2*, and the role of various factors such as time and distance in the presence/distribution of *Xp*. It is necessary to determine the relative fitness of *xopJ2* to derive the resulting selection pressure for retaining the gene in *Xp* and to predict the change in its frequency over time. The objectives of this study were to (i) develop and validate an *Xp* dispersal model under field conditions; (ii) examine the role of *xopJ2* in dispersal velocity of *Xp* in tomato field; (iii) quantify the magnitude of the fitness advantage provided by the gene; and (iv) to determine time of extinction of strains lacking *xopJ2* gene (i.e., *xopJ2* fixation in *Xp* gene pool) in a simulated closed system. The effects of many T3SEs are known in terms of predicted function, avirulence reaction, host range determination, and *in‐planta* growth (Dean, 2011). However, the extent to which a single gene can influence the fitness of bacterial plant pathogens under natural conditions remains poorly explored. Understanding the contribution of XopJ2 to fitness will help to explain its role in evolution of *Xp* and its apparent fixation in the Florida *Xp* population. Fundamentally, an understanding of conserved genes will help in deciphering bacterial evolution.

## Results

### The final *Xp* dispersal model

A binary logistic regression model was developed for predicting the spatiotemporal dispersal of *Xp* using the data collected from 2016 and 2017 trials (Fig. S8‐1). Presence of bacteria was used as the response variable, and field and weather factors were used as independent predictors. The time‐lag period for weather factors was set at 3 days (Table S9‐1, Fig. S9‐1). Out of the 10 main predictors and 16 interactions tested in the full model, only 6 predictors and 4 interactions were significant and sufficient in determining the presence of *Xp* in the final model (Table 1). The stepwise reduction of non‐significant factors was done based on Wald's Chi‐square values and the values for the predictors in final model are presented in Table S9‐1. The Bayesian information criterion (BIC) of the final model was 2410.2, which was reduced from the full model that had a BIC of 2499.9 (Table 2) (see Supporting Information S3 for more information of Wald's tests and BIC).

**Table 1:** Results for fixed predictors for the final logistic generalized liner mixed model developed to predict the spatiotemporal distribution of *Xanthomonas perforans* in tomato field.

Variable	Estimate	SE	*z* value	Pr(>|*z*|)
(Intercept)	−5.676	1.367	−4.151	<0.001
Time (week)	1.258	0.088	14.325	<0.001
Distance (m)	−1.112	0.126	−8.850	<0.001
Gene_XopJ2+	−3.602	0.653	−5.516	<0.001
Temp.mean	−2.117	0.334	−6.343	<0.001
Humidity.mean	1.397	0.303	4.619	<0.001
Rain.sum	−0.203	0.037	−5.447	<0.001
Time:Gene_XopJ2+	0.849	0.122	6.947	<0.001
Distance:Gene_XopJ2+	0.508	0.102	4.978	<0.001
Gene_XopJ2+:Humidity.mean	1.138	0.291	3.912	<0.001
Gene_XopJ2+:Temp.mean	−1.205	0.333	−3.618	<0.001

Notations: Estimate: Coefficient of regression; SE: Standard error; Pr(>|z|): *p*‐value (marginal significance) or *z*‐value; Time: Weeks post inoculation; Distance: Distance from point of inoculation in meters; Gene_XopJ2+: Presence of *xopJ2* gene; Temp.mean: Average weekly temperature (standardized unit); Humidity.mean: Average weekly relative humidity; Rain.mean: Total weekly precipitation.

**Table 2:** Model selection parameters for null model, main effect model, final model, and full model developed to predict the spatiotemporal distribution of *Xanthomonas perforans* in tomato field.

	Null model	Main effects model	Final model	Full model
Number of parameters	2	11	15	39
AIC	4416.92	2440.67	2315.37	2253.82
BIC	4429.55	2510.17	2410.14	2503.04
Log likelihood	−2206.46	−1209.34	−1142.69	−1089.53
Deviance	4412.92	2418.67	2285.37	2186.08
LR Chi‐sq		1994.24	133.30	99.29
DF (LR Chi‐sq.)		9	4	24
Pr(>LR Chi‐sq.)		<0.001	<0.001	<0.001

Notations: AIC: Akaike Information Criterion; BIC: Bayesian Information Criterion; LR Chi‐sq: Likelihood Ratio chi squared value; DF: Degree of freedom; Pr(>LR Chi‐sq): *p*‐value (marginal significance) for likelihood ratio.

Weather predictors dropped from the full model, such as wind speed, were also significant in determining the presence of bacteria but were dropped in the final model as they were highly collinear (*r* > 0.7) to other predictors. There was no significant difference between the two strains (GEV872 and GEV1001) in the presence of bacteria. Based on the model, the interactions of presence of *xopJ2* gene to weeks post inoculation (Time:Gene_XopJ2+) and to distance from point of inoculation (Distance:Gene_XopJ2+) were significant in determining presence of *X*. *perforans* (Table 1). The positive values of estimates for both interactions indicate that XopJ2+ is more likely to be present in the field than XopJ2− (i) as time increases, and (ii) as distance from the point of inoculation increases. As the XopJ2− mutants and XopJ2+ are isogenic strains except for the loss of function of the *xopJ2* gene, the results suggest that the presence of *xopJ2* gene is associated with increased dispersal velocity of *Xp*. The positive estimate for interaction between presence of *xopJ2* and average relative humidity (Gene_XopJ2+:Humidity) strongly supports that there is higher probability of the presence of *Xp* strains with *xopJ2* gene at higher relative humidity whereas the negative estimate of interaction between presence of *xopJ2* and average temperature (Gene_XopJ2+:Temp.mean) indicates that with increase in temperature, the advantage conferred by *xopJ2* gene to the presence of *Xp* is reduced (Table 1). The final model is publicly available at the following repo: github.com/rknx/AvrBst.

### Goodness‐of‐fit and model performance

**Fig. 1:**
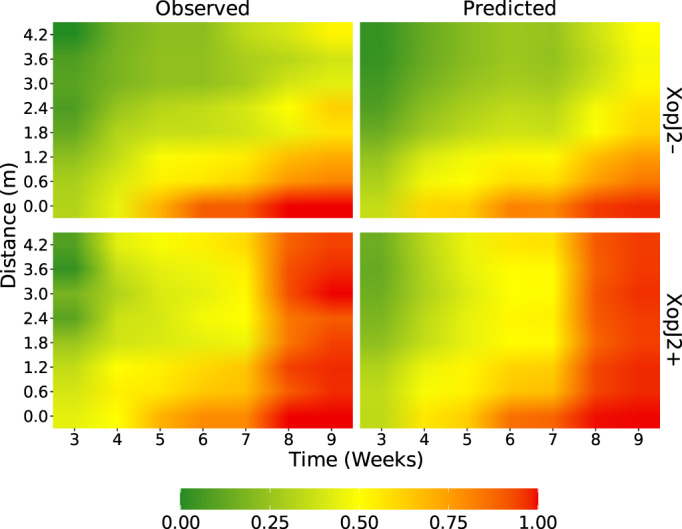
Comparison of average presence of bacteria over distance from point of inoculation and time post inoculation observed in the field to average presence predicted by the final model. Top: in absence of *xopJ2* gene (XopJ2−). Bottom: in presence of *xopJ2* gene (XopJ2+). The predictions were made at same weather conditions as those observed during the experiments.

To determine the goodness‐of‐fit of the model, we first generated a fidelity plot that compares the fitted values of the model to the average observed presence of *Xp*. The spatiotemporal distribution of *Xp* in the field predicted by the final model showed high degree of similarity to observed distribution of *Xp* for both years (Fig. 1). The model showed approximately 97.6% concordance between observed and predicted presence of *Xp*, which is the percentage of pairwise comparisons in which the model predicted higher probability of presence when *Xp* was present in the field than when it was absent and vice versa (Table 3). We also calculated several measures of association which check the agreement between the predicted presence (of bacteria) by the model to the observed presence through enumeration of concordant and discordant pairs (Agresti, 2010) (see Supporting Information S4 for the description of concordance and measures of association). The values of Somers' D, Goodman‐Kruskal's *γ*, Kendell's *τ*_*A*_ and Stuart's *τ*_*C*_ for final model were equal to 0.952, 0.952, 0.476 and 0.951, respectively. The observed and predicted presence of *Xp* were also highly correlated to each other with Spearman's rank correlation coefficient of 0.824 and point biserial correlation of 0.872 (Table 3) (Tate, 1954; Spearman, 1987). The values for coefficient of determination show that the fixed predictors explained approximately 58.4% of the variance in the observed presence of *Xp* (marginal *R* ^2^), whereas, together with random factors, the model accounted for 85% of total variance (conditional *R* ^2^) (Table 3) (Nakagawa *et al*., 2017).

**Table 3:** Goodness‐of‐fit parameters for final model developed to predict the spatiotemporal distribution of *Xanthomonas perforans* in tomato field.

Statistic	Value
Concordance	0.976
Discordance	0.024
Tie	2.4 × 10^−6^
Number of pairs (mil)	4.191
Somers' *D*	0.952
Goodman–Kruskal's Gamma (*γ*)	0.952
Kendall's Tau *A* (*τ*_*A*_)	0.476
Stuart's Tau *C* (*τ*_*C*_)	0.951
Spearman's rho (*ρ*)	0.824
Point biserial correlation	0.872
*R* ^2^ (marginal)	0.584
*R* ^2^ (conditional)	0.850

**Fig. 2:**
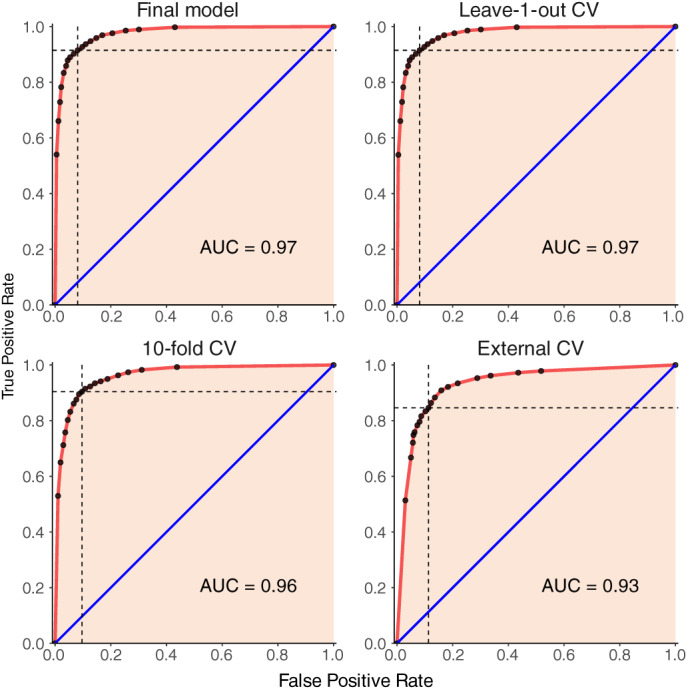
Receiver's operating characteristics (ROC) curve showing the Area under curve (AUC) for final and cross validation models. Top‐left: Final model, Top‐right: Leave‐1‐out cross validation model, Bottom left: 10‐fold cross validation model, Bottom right: External cross validation model using Fall 2015 dataset. Black dots represent the true positive rate (TPR) and corresponding false positive rate (FPR) at various cut‐off intervals. The red ROC curve was generated by smoothing over the black dots. Blue line represents prediction expected by random chance (TPR = FPR). Dashed horizontal and vertical line represent TPR and FPR, respectively, at cut‐off threshold of 0.5.

A ROC curve generated for the final model had an area under ROC curve (AUC) approximately equal to 0.97 (Fig. 2) (see Supporting Information S5 for description of ROC and AUC), where a value greater than 0.9 indicates an excellent predictive performance (Allouche *et al*., 2006). The balance between true positive rate (TPR) and true negative rate (TNR) was maximized at the discriminant threshold of *p* = 0.5. At that threshold, a confusion matrix was built, and several metrics of binary classification were calculated (Liu *et al*., 2011) (see Supporting Information S6 for description of confusion matrix and binary classification metrics). The final model showed a high ability to distinguish presences from absences, with both sensitivity and specificity larger than 91.5% (Table 4). The model was able to correctly predict 91.7% of the observations from field samples. The values of Kappa, Matthews correlation coefficient (MCC), and True Skill Statistic (TSS) were all approximately equal to 0.834, where a value greater than 0.8 represents an excellent model for predictive purposes.

**Table 4:** Model performance parameters for final and cross‐validation models at threshold cutoff of *p* = 0.5.

Test statistic	Final model	Leave‐1‐out CV	10‐fold CV	External CV
Sensitivity	0.915	0.915	0.912	0.846
Specificity	0.919	0.919	0.911	0.895
Correct class (Accuracy)	0.917	0.917	0.912	0.864
Cohen's kappa (*κ*)	0.834	0.833	0.823	0.719
Matthew's correlation coefficient (MCC)	0.834	0.833	0.823	0.724
True skill statistic (TSS)	0.834	0.833	0.823	0.741

Notation: CV: cross validation.

### Cross validation

The final model was internally and externally cross‐validated to identify potential overfitting and to determine the independence of the model from the dataset used to generate the model. Internal cross validations (ICVs) were performed by repeatedly taking a random fraction of the dataset from the final model to make a new model and predicting probability of presence of *Xp* for the other fraction of data (see Supporting Information S7 for further explanation of cross validation approaches). Parameter values for classification accuracy obtained from both ICVs, leave‐1‐out ICV and 10‐fold ICV, were close to those obtained from the final model. The sensitivity and specificity were slightly above 90% (Table 4). Consequently, *κ* statistic and TSS did not show much divergence from the final model. The AUC value was approximately equal to 0.97 and 0.96 for leave‐1‐out and 10‐fold cross validations, respectively, which were also close to the AUC of the final model (Fig. 2). The external cross validation (ECV) was performed by predicting the presence of *Xp* for the 2015 data, which was not used to prepare the final model. The results diverged slightly from the final model, which is not unexpected for ECVs. The value of AUC for the ECV was 0.93 (Fig. 2). At discriminant threshold of *p* = 0.5, the sensitivity and specificity were 0.846 and 0.895, respectively (Table 4). The TSS reduced from 0.834 in the final model to 0.741 in the ECV; however, this value was still high enough to show that the model can make good predictions in conditions not used to develop the final model. The results of ICV demonstrated that the final model was not overfitted. The results of ECV displayed relative independence of the model to the training data.

### *xopJ2* is associated with faster dispersal velocity of *Xp*

**Fig. 3:**
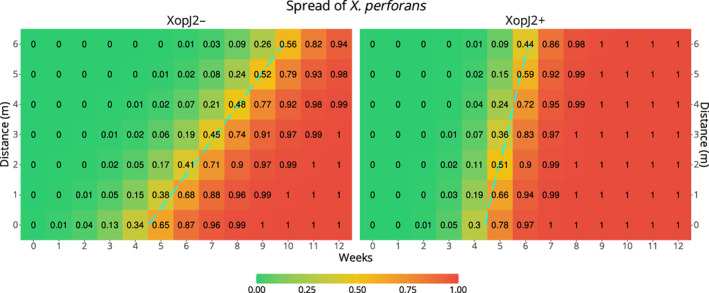
Presence of bacteria predicted by the model over distance and time. Left: when *xopJ2* gene is absent, right: when *xopJ2* gene is present. Blue dashed line traces probability of presence is 50% (*p* = 0.5). The presence of bacteria was calculated at uniform weather condition equal to mean observed during the experiments.

The Time:Gene_XopJ2+ and Distance:Gene_XopJ2+ interactions were significant in the final model, meaning the presence of *xopJ2* gene altered the dispersal velocity of bacteria. The absolute dispersal velocity varied according to the weather conditions, however, in all cases, the ratio of the change in dispersal velocity of *Xp* due to presence of *xopJ2* remained constant as there was no significant interaction between Distance and Time, and between Distance or Time and weather predictors (Table 1, also see Supporting Information S12). Applying the values of estimates from Table 1 in Eq. 2, the dispersal velocity of XopJ2+ was determined to be 3 times that of XopJ2−. The presence of bacteria at a series of distances from point of inoculation and time post inoculation was predicted under a uniform weather condition set at weekly averages that were observed during experimental periods (Table S13‐1). At those conditions, the dispersal velocity of wild‐type XopJ2− bacterium was calculated to be 1.15 m/week, whereas that of the mutant XopJ2+ bacterium was about 3.5 m/week (Fig. 3). It should be noted that these values do not correspond to the increase in the probability of finding a particular gene‐type of *Xp* at a fixed spot, but rather represents the change in maximum distance covered by the bacteria over time at a constant decision boundary.

### *xopJ2* is associated with increased field area coverage of *Xp*

**Fig. 4:**
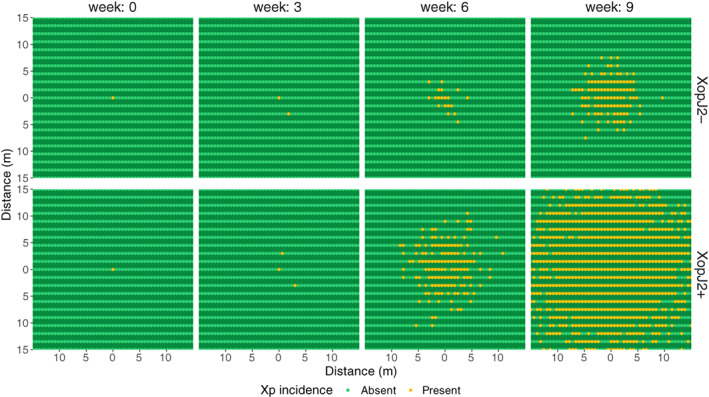
Distribution of *Xp* in a 30 m × 30 m field simulated based on the final model at various time post inoculation. Top: when *xopJ2* is absent, Bottom: when *xopJ2* is present. Black dots represent the central inoculated plants.

**Fig. 5:**
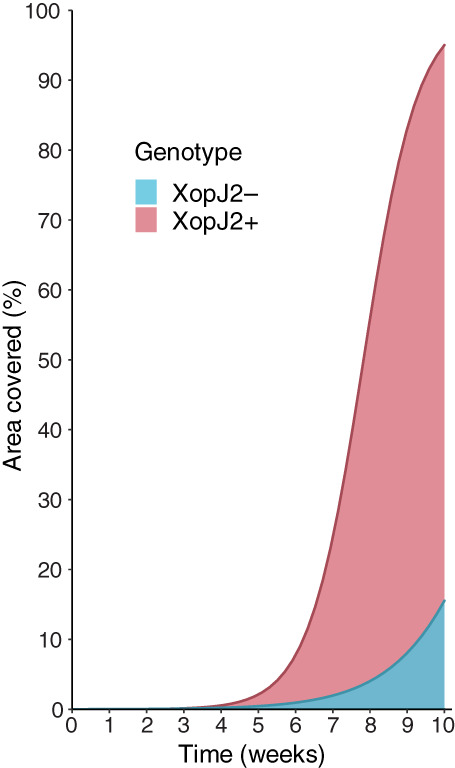
Change over time in area of the simulated field covered by *Xp* when *xopJ2* is absent (XopJ2−) or present (XopJ2+). Cut‐off threshold of 0.5 was used for presence of *Xp*.

The distribution of XopJ2+ and XopJ2− was simulated using the final model at a uniform weather. Under those conditions, the higher dispersal velocity of XopJ2+ translated to a larger area of distribution of *Xp* strains with *xopJ2* than the strains lacking the gene at any timepoint following initial inoculation. The comparison of the distribution of XopJ2+ and XopJ2− in a simulated field showed contrasting differences in dispersal velocity (Fig. 4). By the time XopJ2+ covered almost the entire 30 m × 30 m simulated field, XopJ2− strains were dispersed to approximately 5 m from the point of origin. We also calculated the fraction of area of the field covered by *Xp* at different timepoints following inoculation. At 8 weeks post inoculation, area covered by XopJ2+ was approximately 9.3 times larger than the area covered by XopJ2− (Fig. 5).

### *Xp* strains carrying *xopJ2* increase in frequency over generations of tomato

**Fig. 6:**
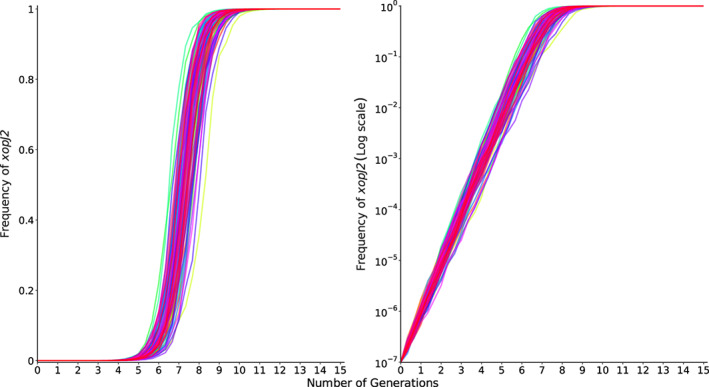
Prediction of frequency of strains possessing *xopJ2* over generations of tomato crop because of selection pressure. Left: frequency in linear scale; Right: frequency in log scale.

In a simulated closed system where there is no entry of *Xp* from outside the field, the relative proportion of *Xp* strains in subsequent generation is proportional to the area covered by the strains in the previous generation. Due to differences in their distribution in field, XopJ2+ bacteria are almost 9.3 times as likely to be transmitted to the next cycle of the crop as XopJ2− bacteria under an assumption of a closed system. Therefore, *xopJ2* increased relative fitness of *Xp* by 9.3 times. The change in frequency of *xopJ2* gene over multiple cropping seasons of tomato was determined from a simulated competitive assay with selection and random drift, but not mutation and migration. The fitness difference among strains with and without the gene led to exponential growth of *Xp* strains with *xopJ2* in the population (Fig. 6). In the simulation, it took 8 to 10 seasons of successive tomato crops after first detection for the frequency of *xopJ2* to reach 75% and another two seasons for its fixation in the *Xp* gene pool (Fig. 6).

## Discussion

HGT is an important component of bacterial evolution (Newberry *et al*., 2019). Like other bacteria, *Xp* has evolved through acquisition of foreign genes (Timilsina *et al*., 2019). The role of horizontally transferred effector *xopJ2* in *Xp* is perplexing. The effector, *xopJ2* is a major elicitor that prevents many strains of *Xp* from infecting pepper. Furthermore, like some other members of XopJ family, *xopJ2* does not seem to significantly change *in‐planta* population size (Noël *et al*., 2003; Abrahamian *et al*., 2018). Maintaining such genes without any apparent selective advantage comes at a fitness cost; hence bacteria tend to lose superfluous genes through the process of genetic decay (Koskiniemi *et al*., 2012; Barak *et al*., 2016). Retention and even selection of *xopJ2* in *Xp* despite the fitness cost must be due to some other fitness factor not related to *in‐planta* population size. In this study, we demonstrated that the fitness associated with *xopJ2* is due to increased dispersal velocity of *Xp*. Increased dispersal velocity leads to a more rapid and widespread distribution, which means that the *Xp* with *xopJ2* can establish earlier in healthy leaves, potentially multiply to a larger population size across a tomato field and be more likely to be transmitted to the next crop cycle. However, XopJ2+ strains did not inhibit further spread of XopJ2− strains. We did not find any *in‐planta* competition or antagonistic activity associated with presence of *xopJ2* gene as XopJ2− distribution increased eventually in plants that were already colonized by XopJ2+ (Fig. S14‐1). This was also evident from previous studies on in‐planta population level of *Xp* and *Xe* (Szczesny *et al*., 2010; Abrahamian *et al*., 2018).

The model generated in this study has high classification accuracy, i.e., the ability of a model to set a boundary that can clearly distinguish negative results (absence of *Xp*) from positive ones (presence of *Xp*). The model accuracy parameter values obtained from ECV were slightly lower than those from the final model. There are three possible reasons for this difference: (i) ECV cannot take advantage of the random covariates; (ii) the tomato variety as well as inoculation method used in Fall 2015 trail (FL47, infiltration of 4 leaves) were different from those in Spring trials (HM1823, dip inoculation) that were used to prepare the model; and (iii) the field data were collected only up to 7 weeks post inoculation (wpi) rather than 9 wpi, and the model better predicts positive presence towards final weeks (resulting in lower sensitivity). The fall weather conditions in the external data sometimes fell outside the range of the weather used in the model, possibly leading to reduction in accuracy of the prediction by the model (Fig. S13‐1). Thus, predictions made using this model are more accurate within or close to the range of weather conditions observed during the spring trials (Table S13‐1).

While the dispersal model generated in this study has a high predictive performance, limitations in its application may arise from the fact that species‐specific, and even strain‐specific differences in the fitness conferred by *xopJ2* are not known outside of two strains of *Xp* tested. Kim *et al*. (2010) reported an increase in *in‐planta* population of *Xe* due to *xopJ2* but Szczesny *et al*. (Szczesny *et al*., 2010) did not find any significant differences. It is possible that the discrepancies are of host origin. The comprehensive role of *xopJ2* in the genus *Xanthomonas* can only be determined from a larger scale study encompassing more strains, multiple species, and several host varieties. The model may also suffer from bias due to low sampling rate per leaf sample. The dataset for the model was generated by examining only 4 colonies from each leaf sample, so there is a high probability of not detecting XopJ2+ or XopJ2− due to sampling bias. At equal population of XopJ2+ and XopJ2−, there is 12.5% chance of not detecting one of the gene‐types. If XopJ2− population is 1/5^th^ of total population in that sample because of its delayed dispersal, then the chance of not detecting it by random sampling is 40%. Nonetheless, the high number of replications in the experiments (40 samples for each distance and time post inoculation) should significantly lower the probability of not detecting strains with either allele of *xopJ2*.

The previous work by Abrahamian *et al*. (2018) studied the maximum distance at which *Xp* could be recovered at different timepoints. One of the shortcomings of the study was that the maximum recoverable distance was limited to the most distant observation possible from the point of inoculation, so the true extent of difference in dispersal between *Xp* strains with and without *xopJ2* could not be discerned. The model in this study used the presence of *Xp* as response variable rather than the distance of dispersal. This allowed the model to predict the trend of *Xp* dispersal (and the difference between XopJ2+ and XopJ2−) outside the maximum distance observed in the field based on the change in *Xp* presence over time within the observed distance. In addition, this enabled us to use data collected from all plants in the field rather than just the maximum distance at each timepoint. Besides, the model used time post inoculation as a numerical variable rather than a categorical variable. The latter allowed us to (i) determine the linear effect of time, and (ii) include weather variables to account for other variances in *Xp* presence over time. Thus, this modelling framework enables model generated here to be more robust in predicting *Xp* distribution and extends findings beyond work conducted by Abrahamian *et al*. (2018). In addition, a single model was presented for both strains used, GEV872 and GEV1001, and it is determined that there was no significant difference between these strains. Finally, the model was validated, and results showed that the model has good performance based on parameters estimated. This enabled us to use it for further additional applications such as determining the dispersal velocity of *Xp* and extinction of *Xp* strains lacking *xopJ2*.

The analytical approach and codebase used for generating this model can serve as a framework for similar studies on the effect of effectors involved in other pathosystems. The steps are designed to be modular and efficient in terms of computing resource. There are provisions for adjusting the steps depending on the requirement of the researcher. Some of the features include auto scaling and normalization of data for improved gradient descent, grouping and filtering of collinear predictors for factor reduction, determination of time‐lag by multiple approaches, reproducibility, and highly readable code. Scripts in R language are provided for cross‐validation (including parallel processing codes for high‐performance computing), goodness‐of‐fit tests and likelihood ratio test for fixed as well as random predictors, The github repository (github.com/rknx/AvrBsT) provides further scripts for animation of spatiotemporal dispersal of pathogen, 3d surface plots generation, confidence interval of dispersal pattern as well as a shiny web‐interface for quick prediction of pathogen distribution based on the model and user provided inputs. These publicly and freely available online resources can be easily adapted to perform analysis and output visualization for studying the effect of bacterial effectors involved in BST or any other pathosystems.

The model developed in this study predicts almost a 3‐fold increase in the dispersal velocity of *Xp* strains when these strains harbour the *xopJ2* gene. We also showed that the difference in fitness resulting from such an increase in dispersal can cause fixation of the gene in a simulated *Xp* population. When the simulation is idealized, it closely aligns with the observed change in the proportion of *Xp* strains containing *xopJ2* in Florida‐wide strain collections between 1998 and 2012. If we consider the year 1998, when *xopJ2* gene was first identified in *Xp*, as generation zero and one generation of tomato crop per year, then our prediction of approximately 75% frequency of *xopJ2* by eighth season corresponds to the 2006 survey result and fixation of the gene in the gene pool in next few years is in agreement with the result from 2012 survey (Timilsina *et al*., 2016). It is important to note that while this model provides empirical support for the hypothesis that the fitness conferred by *xopJ2* gene was a major driving factor for its increased prevalence in *Xp* strains, it does not rule out the effects of other evolutionary forces acting in the *Xp* population, such as the shift from prevalence of T3 strains of *Xp* to T4 strains, changes in distribution of phylogenetic groups or acquisition of other effectors over this period. In addition, both *Xp* strains used in this study were T4 strains and the magnitude of fitness conferred by *xopJ2* in T3 strains might vary from that predicted by the model.

Many other bacterial genes have been studied to understand the fitness that they confer to plant pathogenic bacteria (Vera Cruz *et al*., 2000; Guttman and Greenberg, 2001; Wichmann and Bergelson, 2004). These studies often focus on the effect of the gene on pathogenicity, virulence, aggressiveness, and survival. If the pathogen loses a gene with high fitness advantage, it will become comparatively weaker and is less likely to contribute to the next generation. Consequently, the higher the fitness cost associated with the loss of a gene, the lower the chance of that gene being lost. This study demonstrates that loss of *xopJ2* indeed has a high fitness cost – a 9‐times reduction in relative fitness – and it is indeed conserved in Florida *Xp* population. Hence, incorporation of an R‐gene targeting the avirulence gene *xopJ2* could help in managing bacterial spot of tomato. The gene responsible for *xopJ2*‐mediated HR in pepper has yet to be identified. A *xopJ2*‐mediated HR was also observed in wild tomato, *Solanum lycopersicoides* (Wang, 1992). Transfer of the resistance gene from either source into tomato cultivars would serve as genetic material to develop host resistance against bacterial spot caused by *Xp*.

It is not yet well understood how *xopJ2* enhances dispersal of *Xp*. Since the presence of *xopJ2* does not affect the *Xp* population size *in‐planta*, it must be acting in one or more of the following four stages of disease cycle: egress, transport, survival, or ingress. However, it is unlikely for *xopJ2* to confer fitness during transport or survival in the phyllosphere; as a T3SE, it is directly secreted into the host cell. Several pathogens have been reported to manipulate host water homeostasis to promote water‐soaking and consequently the egress of the pathogen from mesophyll space to the phyllosphere. The T3SE Avrb6 found in cotton pathogen *Xanthomonas campestris* pv. *malvacearum*, was reported to promote water‐soaking symptom in the host, leading to higher population of bacteria released to leaf surface (Yang *et al*., 1994). Water‐soaking was also promoted by another TAL effectors such as AvrHah1 from *X*. *gardneri* and effectors AvrE and HopM1 of *Pseudomonas syringae* pv. *tomato* (Xin *et al*., 2016; Schwartz *et al*., 2017). Many T3SEs have been shown to promote ingress, mostly by disrupting the closure of stomata in response to the bacteria. In *P*. *syringae*, multiple effectors such as HopM1, HopF2, AvrB and HopZ1a can inhibit closure of stomata (Hurley *et al*., 2014; Lozano‐Durán *et al*., 2014; Ma *et al*., 2015; Zhou *et al*., 2015). The effector HopZ1a is a homologue of XopJ2 and shares similarities such as acetylation of host kinases and disruption of microtubules (Lee *et al*., 2012). When XopJ2+ and XopJ2− mutant *Xp* strains were sprayed on tomato, the proportion of leaf area covered by lesion was 2 to 3.5 times higher in WT than in mutant, thus supporting that XopJ2 can increase the efficiency of leaf ingress (Abrahamian *et al*., 2018). Further studies are needed to determine the phase of disease cycle at which *xopJ2* promotes dispersal.

Evolutionary changes, by definition, are in a constant state of change, leading to erosion of existing genes and acquisition of new ones. A recent collection of *Xp* strains from tomato fields revealed a new phylogenomic group of *Xp* strains that lack *xopJ2* (Klein‐Gordon *et al*., 2020). Half of those strains were found to carry a novel homologue of *xopJ2*. Mutually exclusive presence of *xopJ2* and the new homologue in separate clusters was also reported in Alabama and Florida *Xp* population (Klein‐Gordon *et al*., 2020; Newberry *et al*., 2020). This new homologue shares approximately 71% nucleotide identity with *xopJ2* and elicits HR when inoculated in pepper. It is not known if it serves the same fitness purpose as *xopJ2* in terms of dispersal. More large‐scale surveys will reveal if this new homologue is gradually replacing *xopJ2* from the *Xp* gene pool. In any case, *xopJ2* is still widely prevalent in *Xp* population in Florida tomato fields just like our model predicted. This study shows that a single bacterial gene is capable of driving evolutionary forces that alter the structure and distribution of the bacterial population leading to its fixation in the gene pool.

## Experimental procedures

### Data generation

The approach used for data generation, including bacterial strain selection, mutagenesis, field trials (inoculation and data collection), and identification of recovered strains was as described previously by Abrahamian *et al*. (2018). Briefly, the functional *xopJ2* gene was partially deleted to generate near‐isogenic mutants with non‐functional *xopJ2* gene (XopJ2−) in two wild‐type (XopJ2+) *Xp* strains, GEV872 and GEV1001. Equal concentration of XopJ2+ with respective XopJ2− was co‐inoculated into the central plant in a linear row of 15 tomato plants, with five replications each for strains GEV872 and GEV1001, separately (Fig. S8‐1). The experiment was repeated in three experimental periods: fall 2015, spring 2016 and spring 2017. Leaf samples were collected every week from the young flush of each plant between 3–9 wpi in spring trials and 3–7 wpi in the fall trial. The leaf washings of each sample were plated on agar plates and colony forming units (CFU) as well as the presence of XopJ2+ or XopJ2− bacteria was determined. For each growing season, the weather data, including temperature, rainfall, relative humidity, dewpoint, wind velocity, and solar radiation, were downloaded from Florida Automated Weather Network (fawn.ifas.ufl.edu).

### Data preprocessing

In our analysis, XopJ2+ was indicated to be present in a tomato plant if at least one bacterial colony isolated from the leaf samples from that plant tested positive for the full length *xopJ2* gene in the current sample collection or any previous samples from the same plant. For XopJ2−, we used the same set of criteria except that positive detection of the partially deleted *xopJ2* gene was required. Samples in which bacteria colonies were obtained but XopJ2+ or XopJ2− could not be determined were indicated as missing values.

The best ‘time‐lag’ period for weather variables, which is the amount of time required for the effect of the weather to become detectable at the time of bacterial isolation, was determined for weather parameters using the methods described in Supporting Information S9 (van der Plank, 1963). Records for each weather variable were scaled (*z*‐score normalization) to improve computational performance of gradient descent during regression (Gelman and Pardoe, 2007). Weekly averages of weather records including daily minimum, daily maximum, daily average/total, and day (7 am to 9 pm) and night averages were calculated accounting for the time‐lag and added to field data from the respective week.

### Statistical analysis

All statistical analyses were performed in r v3.6 (R Foundation for Statistical Computing, Vienna, Austria). Models were developed using Generalized Linear Mixed Model (GLMM) using lme4 package (Bates *et al*., 2014). Wald's chi‐squared test was performed using car package (Fox and Weisberg, 2018a). In‐house scripts were developed for calculating goodness‐of‐fit and model accuracy parameters, for generating receiver operating characteristic (ROC) curves, and for cross validation. Massively parallel cross validations were performed in HiperGator 2.0 supercomputer at the University of Florida using parallel program (Tange, 2011). The scripts used for this paper are publicly available in snapshot branch of github.com/rknx/AvrBst.

### Model preparation

The model was developed using only the data from 2016 and 2017 spring trials. The 2015 trial was omitted because the field observations were made only up to 7 wpi in this trial as opposed to 9 wpi in other trials. Fixed field predictors consisted of *xopJ2* gene‐type (XopJ2+ or XopJ2−), distance from point of inoculation, number of weeks post inoculation, and the strain of *Xp* (GEV872 or GEV1001). Fixed weather predictors consisted of weekly summaries of weather records described previously. To deal with high collinearity of some weather variables, the weather data were clustered into groups based on correlation and only the best member of each group was used in developing final model (see Supporting Information S9). The full model consisted of all possible interactions between fixed field factors and interactions between gene‐type and weather factors. Experimental season (year), replication, and individual plants were used as random covariates in hierarchical nested form in the full model. Directionality of spread (‘up’ or ‘down’ from central inoculated plant) was also used as a random effect because the preliminary observation showed difference in dispersal in two directions.

### Model reduction

Since default optimizers led to non‐convergence in the full model, a relatively slow iterative algorithm, BOBYQA, was used to facilitate model convergence in all subsequent steps (Powell, 2009). From the full model, non‐significant predictors and interactions were removed in a stepwise manner, starting with the interactions using drop1 function in lme4 package. Significance of the effect of fixed predictors was tested using Wald chi square (*Χ* ^2^) test (both type II and type III, described in Supporting Information S3) and non‐significant (*p*‐value > 0.05) predictors were removed in stepwise manner (Wald, 1943; Fox and Weisberg, 2018b). Among several reduced models, the best model was selected based on BIC (Schwarz, 1978) and the log‐likelihood. Complexity of the models were also accounted for during model selection with the objective of reducing overfitting. Random covariates were selected based on Likelihood Ratio (LR) by comparing model to simpler models or null model with constant random effect (Galwey, 2014) (see Supporting Information S3–S11).

### Goodness‐of‐fit of model

The fidelity plot was generated from observed presence of *Xp* and the fitted values from the model by averaging all presences over distance and time for XopJ2− and XopJ2+ separately. The concordant, discordant and tied pairs were determined by comparing the predicted values from observed presences to those from observed presence of *Xp* to check whether the model predicted higher values when *Xp* was present than when it was absent. The measures of ordinal association such as Somers' D, Goodman‐Kruskal's Gamma (*γ*), Kendall's Tau *A* (*τ*_*A*_) and Stuart's Tau *C* (*τ*_*C*_) were calculated from the table of the paired comparisons (Kendall, 1938; Stuart, 1953; Goodman and Kruskal, 1954; Somers, 1962) (see Supporting Information S4 for further explanation of each measure). Spearman's rho (*ρ*) was calculated as a measure of rank correlation using building cor function in R (Spearman, 1987). Point biserial correlation between binary observation and ordinal prediction was also calculated as previously described (Tate, 1954). Theoretical pseudo *R* ^2^ values were calculated to derive the fractions of the variance of dependent variable that were explained by fixed and all independent predictors as described in Nakagawa *et al*. (2017).

### Assessing model performance

The ROC curve was generated for the final model by plotting the TPR with corresponding False Positive Rate (FPR) at a series of incremental cut‐off thresholds between *p* = 0 and *p* = 1 (Fig. S5‐1). The area under ROC curve (AUC) was determined by integration of all areas under the ROC curve between each consecutive points of the ROC graph (see Supporting Information S5 for further description). An optimal discriminant threshold was selected from the ROC curve by maximizing TPR – FPR, and thus balancing sensitivity with specificity. A confusion matrix was generated by comparing the dichotomously classified values of predicted presence to actual observed presence of *Xp*. Correct classification, Cohen's Kappa (*κ*), MCC and TSS (Cohen, 1960; Matthews, 1975; Allouche *et al*., 2006) were calculated from the confusion matrix (see Supporting Information S6 for description of the terms). TSS was ultimately used as the measure of predictive accuracy as it is independent of prevalence (Allouche *et al*., 2006).

### Model validation

For ICV, leave‐10‐out and 10‐fold approaches were used. In both methods, the model frame (dataset from which the model was developed, excluding the missing values) was divided into training and test sets. In case of leave‐10‐out, 10 randomly selected datapoints were assigned as the test set and the rest of the datapoints were assigned to the training set. In 10‐fold approach, one‐ten^th^ of the entire model frame was randomly selected and used as test set and the rest of the datapoints were used as training set. A new model was developed by using only the training set and the response (presence of bacteria) was predicted for each datapoint in the test set using the predictor values of that datapoint. For both ICV approaches, the process of generation of random pairs of training and test datasets, fitting of training set and prediction for test set was repeated until the prediction was done for all datapoints in the dataset. For ECV, a dataset from fall 2015 experiment was used as test set. The dataset was transformed in similar manner as the original dataset used for modelling. The final model was used to predict response variable using independent predictor values from the test set. Once the predictions were completed for all datapoints for all ICVs and ECV, the predicted presence was used to generate ROC curve and to compute binary classification metrics.

### Dispersal velocity

Dispersal velocity was calculated for mean weather conditions from the final logistic model.

Let *x* ^*t*^ and *x* ^*d*^ be predictors that have significant interaction with weeks post inoculation (*time*) and distance from point of inoculation (*dist*), respectively. At a constant probability (*p*), constant weather conditions, and no *time:dist* interaction, all terms in logistic equation become constant except those containing *time* and *dist* will be constant (see Supporting Information S12 for the detailed derivation). In such a case, *dist* can be calculated as,

(1) $$\text{dist}=\left(-\frac{\beta_{\text{time}}+\sum \left(\beta_{\text{time}:x^{t}}\times x^{t}\right)}{\beta_{\text{dist}}+\sum \left(\beta_{\text{dist}:x^{d}}\times x^{d}\right)}\right)\times \text{time}+\left(\frac{\text{constant}}{\beta_{\text{dist}}+\sum \left(\beta_{\text{dist}:x^{d}}\times x^{d}\right)}\right)$$

which is in the form *y* = *mx* + *c*, where the slope (*m*) represents distance changed per unit time, and hence is the dispersal velocity (*V*) of the pathogen. If gene‐type (*gene*) has significant interaction with *dist* and *time* only, and absence of gene is set as base level or 0 and presence of gene is set at 1 in the logistic regression, the ratio by which the *gene* increases the dispersal velocity can be expressed as:

(2) $$\frac{V_{\text{gene}+}}{V_{\text{gene}-}}=\left(\frac{\beta_{\text{time}}+\beta_{\text{time}:\text{gene}+}}{\beta_{\text{time}}}\right)\times \left(\frac{\beta_{\text{dist}}}{\beta_{\text{dist}}+\beta_{\text{dist}:\text{gene}+}}\right)$$

In such a scenario, a positive estimate for *time:gene* interaction or negative estimate for *dist:gene* interaction for XopJ2+ would indicate that presence of *xopJ2* gene is associated with the increased dispersal velocity of *Xp*.

### Simulation of distribution and selection pressure

The distribution of XopJ2+ and XopJ2− bacterial strains was simulated in a 30 m × 30 m field. The coordinates of tomato plants in the field were determined using row‐row distance (*R*) of 1.5 m and plant–plant distance (*P*) of 0.6 m, with the origin at the centre of the field. The dispersal of bacterial strains was initiated at the centre of the field. The probability of the presence of *Xp* was predicted using the final model in all plant coordinates in the field over time assuming constant mean weather conditions. The presence (present/absent) of *Xp* in a tomato plant was determined based on the probability of presence predicted by the model using rbinom function. The fraction of the field area covered by *Xp* was calculated by simply dividing the number of plants in which *Xp* was present by the total number of plants (1000). The area of field covered by the XopJ2+ and XopJ2– was calculated over time. The relative finesses of the XopJ2+ and XopJ2− were set based on the ratio of the field area covered by them under the simplifying assumption that the inoculum load of bacteria for the future crops is derived only from preexisting bacterial population in the field in a closed system, with no introduction of external bacterial population.

### Simulation of gene frequency over cropping seasons

Using the previously calculated relative fitness, the frequency of *xopJ2* gene was simulated in *Xp* population over several cropping seasons of tomato. The effective population was set at 1 × 10^8^ and the initial frequency of *xopJ2* gene was set to 10^−7^ (Bobay and Ochman, 2018) (see Supporting Information S15 for the derivation of the values). During each successive tomato cropping season, the bacterial population was increased by 100 times proportional to their relative fitness. Then, the entire population was sampled randomly without replacement bringing the population down to the effective population size, thus creating a bottleneck and allowing for a random shift in gene frequency. Furthermore, a random multiplier (lognormally distributed, mean = 0, standard deviation = 0.25) was added for both gene‐types during population expansion to simulate season‐by‐season variability. This process of expansion and selection was repeated for several generations until the gene became either lost or fixed in the population gene pool. The simulation process was repeated 100 times.

## Conflict of interest

The authors declare no conflicts of interest.

## Author contributions

Anuj Sharma and Sujan Timilsina designed the experiment. Peter Abrahamian and Gerald V. Minsavage generated the field data. Anuj Sharma performed the statistical analysis with guidance from James Colee and Peter S. Ojiambo. All authors contributed to data interpretation and writing of the manuscript.

## Supporting information


**Appendix** **S1:** Supporting Information.
