# Supplementary material for: Need for speed: bacterial effector XopJ2 is associated with increased dispersal velocity of Xanthomonas perforans
Source: Environ Microbiol. 2021 Jun 8;23(10):5850–65. doi: 10.1111/1462-2920.15541 (PMC8597037; doi:10.1111/1462-2920.15541)
Supplement: Supplementary file 1 — Appendix S1: Supporting Information. [file EMI-23-5850-s001.doc]

# Need for speed: Bacterial effector XopJ2 is associated with increased rate of dispersal of Xanthomonas perforans

Anuj Sharma1, Sujan Timilsina1†, Peter Abrahamian2#, Gerald V. Minsavage1, James Colee3, Peter S. Ojiambo4, Erica M. Goss1,5, Gary E. Vallad2, Jeffrey B. Jones1‡
*^1^ Department of Plant Pathology, University of Florida, Gainesville, FL, USA.*

*^2^ Gulf Coast Research and Education Center, University of Florida, Wimauma, FL, USA.*

*^3^ Statistics Consulting Unit, Institute of Food and Agricultural Sciences, University of Florida, Gainesville, FL, USA.*

*^4^ Department of Entomology and Plant Pathology, North Carolina State University, Raleigh, NC, USA.*

*^5^ Emerging Pathogens Institute, University of Florida, Gainesville, FL, USA.*

† Corresponding author: Sujan Timilsina; sujan.timilsina@ufl.edu

‡ Corresponding author: Jeffrey B. Jones; [jbjones@ufl.edu](mailto:jbjones@ufl.edu)

**Running title:** *xopJ2* is associated with increased *Xp* dispersal

# Keywords: *[Xanthomonas] [perforans] [bacterial spot] [tomato] [effector] [fitness] [dispersal] [distribution]*

Epidemiological studies of pathogen dispersal and disease spread are necessary for disease prediction and forecasting. These studies rely on the development of regression models from observations made in the field/nursery. Not all models are equal, and they should be tested for their goodness-of-fit and predictive power before inferences can be made based on their predictions (Fun*k et a*l., 2019). Unfortunately, simple linear models are often not very useful for biological/ecological data as they make several assumptions that are not valid for such data (Bolke*r et a*l., 2009). More advanced regression models such as generalized linear mixed models (GLMM) have been used in these fields for model development.

# Generalized Linear Mixed Models

GLMM is the extension of generalized linear model that allow the linear predictors to have both fixed and random effects. Fixed effects are constant across the population and the magnitude of the effect is modeled whereas random effect vary across the population, the variance/variability in the population is modeled. GLMM is the combination of two statistical frameworks — generalized linear model to handle non-normal data such as binary outcomes and linear mixed models to handle random effects (Bolke*r et a*l., 2009). Thus, GLMM is suitable for biological/ecological data which are often not normal and affected by many factors that are of no interest to the study by themselves. GLMM can be expressed in matrix notation as:


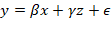
 eq S

1‑

where β is the coefficient of fixed effect x, γ is the coefficient of random effect z, and ϵ is the residual. γ and ϵ are normally distributed with mean equal to 0 i.e., and . Thus, the random effects do not change the expected value, but change the variance of the response variable.

# The Logistic Regression

Logistic regression can be used to model binary or survival response variables, which are extremely common in biological datasets. A binary variable can have one of two values — 0/1 or yes/no or true/false or present/absent. When the response variable is binary, the probability distribution of one of the outcomes (often called success) over multiple trials follows a binomial distribution. Given the probability of success (p) in each trial, the likelihood of success in r out of n independent trials is given by:


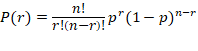
 eq

S2‑

The objective of the logistic regression is to find the probability p. In the most common approach, maximum likelihood estimation (MLE), likelihood () is calculated for a series of probability of success (p) and the final p is determined based on the largest value of i.e., the maximum likelihood.


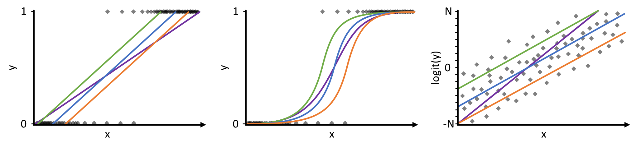


Figure

S2‑1. Diagrammatic representation of modeling of binary variables.

The rhombuses represent individual observations. Left: Linear regression as a bad fit, Center: Sigmoid curves (logistic regression) as a better fit. Right: Logit transformation to convert sigmoid curves into straight lines for regression.

In logistic regression, logit function is used to transform the sigmoid probability distribution into linear line before fitting (Figure S2-1). The logit function is the natural logarithm of the odds. Odds is the ratio of success to failure.


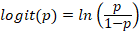
 eq

S2‑

The multivariate logistic regression can be expressed as:


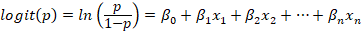
 eq

S2‑

where, β_0_ is the coefficient of the intercept and are the coefficient for dependent variables . The odds of success can be expressed as:


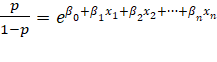
 eq

S2‑

The probability of success (p) can then be expressed as:


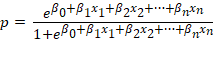
 eq

S2‑

# Significance Test in GLMM

## Wald’s test

The wald statistic can be used to determine the significance of fixed predictors in GLMMs without overdispersion (Wald, 1943). It is calculated by comparing the estimate of the predictors with the standard error. Wald test asymptotically follows a Chi-square (Χ^2^) distribution which is used to calculate the significance of predictors. For models with interaction terms, Wald Χ^2^ values can be type II and type III (SAS notations), and they vary in value and significance for main effects with interactions. The difference between the type II and the type III test is that the type II test preserves the law of marginality i.e., the type III test checks the significance of the predictor in context of all other predictors, whereas the type II test omits higher order interactions of the predictor under test. Wald’s test is unreliable for random effects due to boundary effects, thus increasing type-II errors (Molenberghs and Verbeke, 2007).

## Likelihood ratio (LR) test

LR test determines the contribution of a factor by comparing the deviance models with and without the factor. LR test is useful for selection of random factors in GLMM. Use of LR test for selection of fixed effects may be unreliable as large sample size is needed per level of the effect (Pinheiro and Bates, 2000). In addition to random factor selection, LR test can also be used for model selection between nested models.

## Information criteria (IC)

Information theoretic criteria-based model selection procedures allow comparison among multiple, non-nested models. Akaike information criterion (AIC) and Bayesian information criterion (BIC; also called Schwarz information criterion) are the two most popular IC (Akaike, 1973; Schwarz, 1978). AIC uses deviance of the model as a measure of fit and reports the magnitude of difference in expected predictive power compared to the null model. BIC is a variation of AIC that uses a bayesian approach i.e., MCMC to calculate confidence interval for both fixed and random parameters and also penalizes more complex models (Baaye*n et a*l., 2008).

## Coefficient of Determination (R^2^)

R^2^ is a popular measure of goodness-of-fit for linear models (Draper & Smith, 1998). R^2^ is a summary statistic of proportion of variance in response variable that is explained by independent predictors of a statistical model. Unlike IC, R^2^ is comparable across dataset and gives an absolute estimate of goodness-of-fit. There is no standard way of calculating R^2^ for mixed models; however, several approaches have been proposed (Piepho, 2019). Once such approach proposed by Nakagawa et al. (2017) divides variance components into fixed (), random (), individual-specific (), and residual () variance. The ratio of fixed variance to total variance (sum of all four variances) gives marginal R^2^ whereas the proportion of fixed, random, and individual-specific variance to total variance gives the conditional R^2^. Residual variation has further been divided into overdispersion term () and distribution-specific () variance, where binary logistic regression with logit link has distribution-specific variance of π^2^/3. In summary, for binary logistic regression with logit link, the R^2^ values can be calculated as:


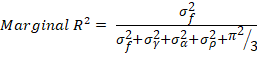
 eq S

3‑


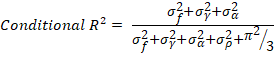
 eq S

3‑

# Measures of association

Association is simply the measure of strength and direction of the relationship between variables. In the context of logistic regression, association is defined as the degree to which the predicted probabilities agree with the actual observation of the response variable. The fitted prediction in the logistic regression is in probability scale whereas the observed response is in binary scale . Measures of association are used to check if higher probability of success is correlated with observed success and vice versa (Denui*t et a*l., 2019).

## Concordance, discordance, and tie

A pair of observations is said to be concordant if the observation with the larger value of the first component has also the larger value for the second component. Concordance and discordance are defined as the fractions of concordant and discordant pairs in all possible combinations of two observations. For two variables x and y, a pair of observations and is said to be concordant if , discordant if and tied if . Various components of the tie are not discussed here.

Often, in a binary logistic regression analysis, comparisons are made between observations with success and those with failure, but not among themselves (as in SAS PROC LOGISTIC). In such a case, a pair of observations is said to be concordant if the predicted probability of success is larger than that of failure. In other words, a paired comparison is said to be concordant if , discordant if and tied if . Then,


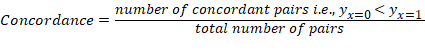
 eq S

4‑


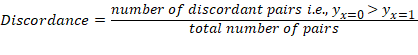
 eq S

4‑


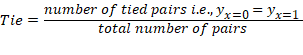
 eq S

4‑

### Kendell’s tauA (**τ_A_**)

Kendell’s τ (or τ_A_ for distinction from other tau statistics proposed later) is simply defined as the difference between number of concordant and discordant pairs expressed as the fraction of total number of pairs compared (Kendall, 1938; Denui*t et a*l., 2019).


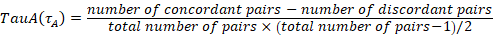
 eq S

4‑

### Stuart’s tauC (**τ_C_**)

τ_C_ (also, Kendell’s τ_C_) is a modification of τ for non-square contingency table (Stuart, 1953).


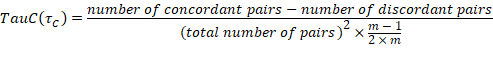
 eq S

4‑

where, m is smallest dimension of contingency table. for binary observation.

### Goodman-Kruskal’s gamma (**γ**)

γ is defined similar to τ, except ties are ignored (Goodman and Kruskal, 1954). γ is considered superior to τ_A_ if there is a large number of ties.


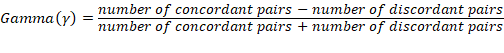
 eq S

4‑

### Somers’ D

D is defined similar to τ_A_, except the ties in independent variable are ignored. Thus, D is asymmetric and often used with regressions only (Somers, 1962).


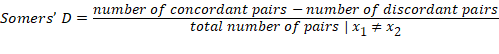
 eq S

4‑

# Receiver Operating Characteristics Curve

Receiver Operating Characteristics (ROC) curve is the graph that illustrates degree of distinction between two categories at a series of discriminant threshold. In case of binary logistic regression model, it is the ability of the model predictions to distinguish between two classes of observations (i.e., success vs failure). As mentioned earlier, the response variable of logistic regression is in binary scale i.e., , but the fitted predictions are in probability scale, i.e., . To access the accuracy of the prediction, y needs to be converted into binary scale. This can be done by cutting off the probability dichotomously at a cut-off threshold, also known as discriminant threshold (Figure S5-2).


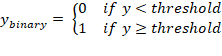
 eq

S5‑

True

negatives

True

positives

False

negatives

False

positives

Distribution of

predictions for

real negative

observations

Distribution of

predictions for

real positive

observations

Threshold

**FPR**

**TPR**

ROC

AUC

Random

Prediction

(TPR = FPR)

**Predicted probability.**

**Number of predictions**

Threshold

Figure

S5‑2. Diagrammatic representation of binary classification. Left: The true positives and false positives at a threshold, Right: Plot of TPR against FPR at a series of thresholds.

The ability of the model to discriminate positive and negative observation correctly and precisely depends on the value of the threshold used. Lower discriminant threshold leads to higher true positive rate (TPR) but also higher false positive rate (FPR) and vice versa.


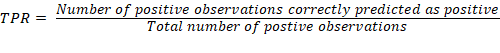
 eq

S5‑


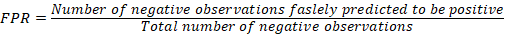
 eq

S5‑

ROC curve is a plot of TPR against FPR at a decreasing series of threshold from 1 to 0. It can be used to identify the most suitable discriminant threshold to maximize the predictive accuracy of the model. In a random prediction, , so ROC curve will be straight line of intercept 0 and slope 1. In models with better than random predictions, TPR increases more steeply compared to FPR when threshold is reduced from 1, but as threshold approaches 0, FPR gradually catches up to TPR.

The performance of distinction can be estimated by calculating the Area under ROC curve (AUC).


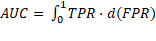
 eq

S5‑

AUC represents the measure of separability of the success from the failure based on the predictions and is equivalent to the probability that prediction for a randomly chosen success is higher than a randomly chosen failure. The interpretations of the AUC values for usability of the model for prediction are as follows: : worse than random, : poor, : useful, : good and : near perfect (note that ‘[]‘ stands for inclusive and ‘()‘ stands for exclusive).

# Binary classification metrics

## Confusion matrix

After a suitable discriminant threshold is identified from the ROC curve, the predicted probability can be converted into binary scale using that threshold as shown in equation S5-. Based on actual observations and binary predictions, a confusion matrix can be generated as follows:

| Observed  Predicted | Success () | Failure () |
| --- | --- | --- |
| Success () | True positives (A) | False positives (B) |
| Failure () | False negatives  (C) | True negatives (D) |

Figure

S6‑3. A typical confusion matrix showing tabulations of agreements between observations and predictions.

## Sensitivity and specificity

Sensitivity is defined as the fraction of observed successes that were predicted as success. Specificity is the fraction of observed failures that were predicted by the model as failure. Sensitivity and specificity are equal to TPR and 1-FPR at the given threshold.


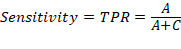
 eq S

6‑


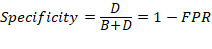
 eq S

6‑

Sensitivity measures how well a model can predict success, whereas specificity measures how well it can predict failures.

## Accuracy

Accuracy or correct classification is defined as the fraction of observations that were predicted correctly. It is the measure of how well the model can correctly predict the observation as success or failure.


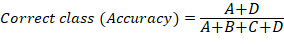
 eq S

6‑

## Cohen’s kappa (κ)

The κ statistic measures the extent to which the agreement between observation and prediction by the model exceeds the prediction by random chance (Cohen, 1960). The degree of agreement indicated by κ values is as follows: : no agreement, : none to slight, : moderate, : substantial, and : near perfect (note that ‘[]‘ stands for inclusive and ‘()‘ stands for exclusive).


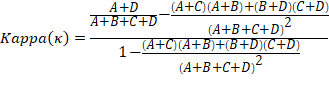
 eq S

6‑

## Matthew’s correlation coefficient (MCC)

The MCC (equivalent to φ coefficient) is a measure of correlation between two dichotomous variables. It has been used widely in the field of machine learning for measuring the strength of relation between input-output pairs (Matthews, 1975; Kraemer, 2006).


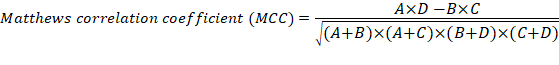
 eq S

6‑

## True skill statistic (TSS)

TSS (identical to Youden's index J) is defined as the average of correct prediction rate for success and failures (Peirce, 1884; Youden, 1950; Allouch*e et a*l., 2006). It is considered the best available summary measure of model performance (Li*u et a*l., 2011). TSS ranges from –1 to +1 and the model is classified as follows based on the TSS value: worse than random, : poor, : useful, : good, : excellent, and : perfect model (note that ‘[]‘ stands for inclusive and ‘()‘ stands for exclusive).


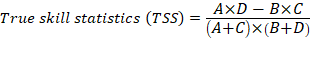
 eq S

6‑

Alternatively,
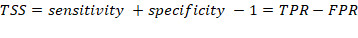
 eq S

6‑

# Cross-Validation

Cross-validation of a regression model is the process of testing its predictive ability for forecasting outside of the data on which it is based on. In addition to that, cross-validation helps to identify signs of over-fitting of the model. There are several approaches to cross validation; however, most approaches rely on model development with a dataset (called training dataset) and evaluation of the model with data not used as part of training dataset (called test dataset). Machine learning often uses an additional dataset called validation dataset to fine-tune the model parameters. Cross-validation is performed to determine the relative usefulness of a model for prediction as well as to identify over-fitting. A regression model can be cross-validated internally or externally.

## Internal cross validation

In this approach, the model frame (dataset from which the final model is developed) is divided into training and test sets. The number of datasets in training and test sets varies according to the approach and is described below. A new model is developed by using only the training set and the response is predicted for each datapoint in the test set using the respective predictor values. The process of generating random pairs of training and test datasets, fitting of training set and prediction for test set is repeated until the prediction is completed for all datapoints in the model frame. After that, various binary classification metrics can be calculated.

### Leave-p-out ICV

In this approach, p number of randomly selected datapoints are assigned as the test set and the rest as training set.

### k-fold ICV

In k-fold approach, one-k^th^ of the entire model frame is randomly selected and used as test set and the rest as training set.

## External cross validation

This approach requires input of a dataset that was not used to prepare the regression model under validation. The external dataset (test set) should have same predictors as model frame (training dataset). Using the independent predictor values from test set on the model, the response variable was predicted for the entire test set. Then, the actual observed values and the predicted values for the response can be compared by calculation of binary classification metrics such as TSS.

# Experimental layout


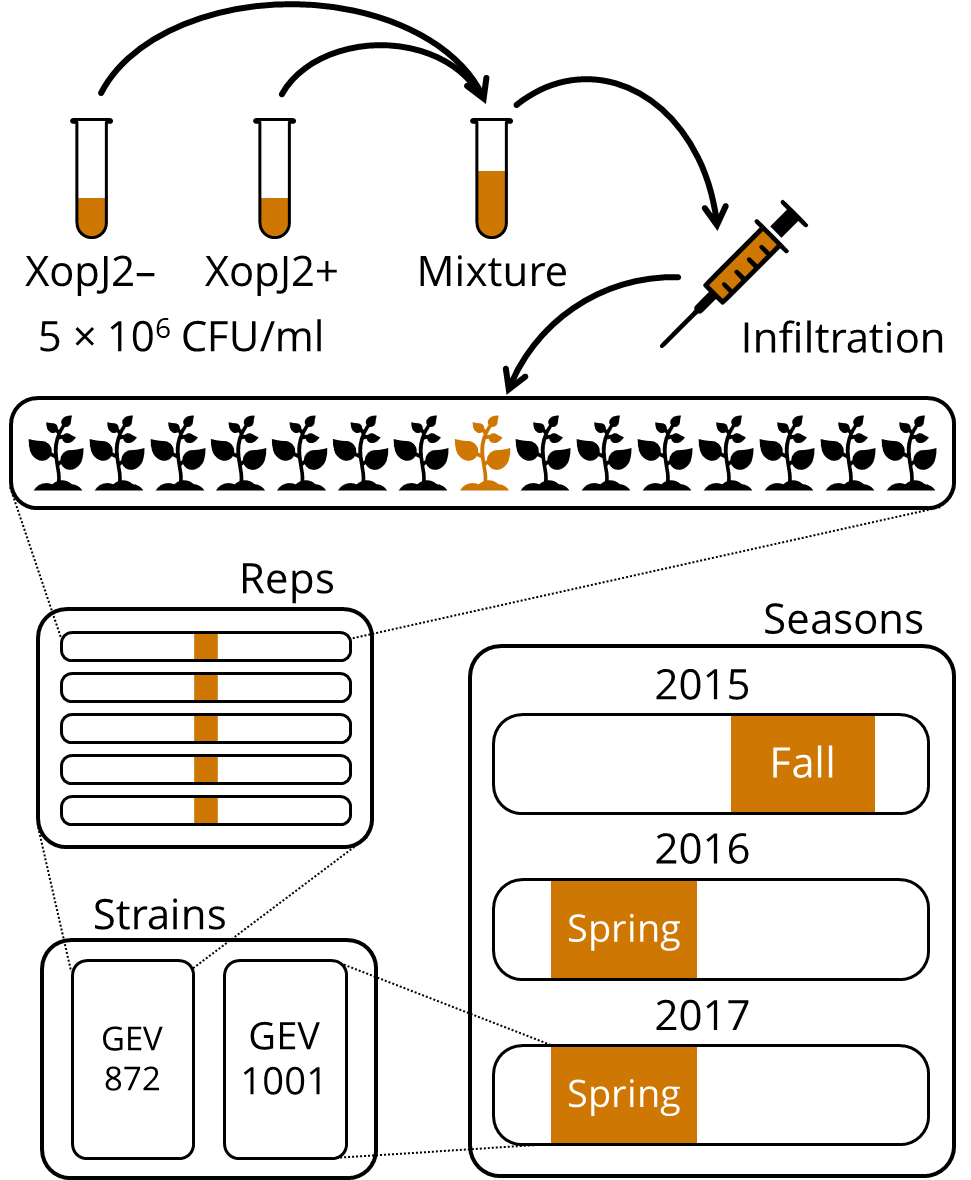


Figure

S8‑4. Experimental design, including inoculum preparation, inoculation, field plot layout used for data generation.

# Time‑lag determination

Time‑lag is the period of the time between the moment an event occurs and the moment when its effects can be detected or measured. In this case, the events are the changes in weather patterns and the effects are the changes in presence of *Xp*. There is no standard method for calculating the period of “time‑lag” for the effect of weather factors on the pathogen population as it is variable between different pathosystems and environmental conditions. Additionally, there is no standard statistical analysis to derive the right time‑lag period (Lecler*c et a*l., 2014). Moreover, this period can be a function of the levels of those factors. For example, environmental temperature close to the optimum temperature for bacterial growth will lead to a more rapid increase in bacterial population and a faster rate of plant metabolism resulting in a smaller time‑lag period. Models that do not account for time-lag tend to underestimate the severity of infection and the progression of disease (Wearin*g et a*l., 2005). Various approaches have been recommended for determining the most suitable time-lag for model development, with the aim to maximize predictive accuracy. In this study, we used a sliding window approach for a lag period of 0 to 6 days between all the weather variables and the detectable change in presence of bacteria.

**Clustering of weather predictors**: As multiple weekly statistics (maximum, minimum, mean etc.) were calculated from each weather variable, clustering of weather predictors was necessary for reducing the number of highly correlated predictors. Although none of the weather variables were truly orthogonal to each other, the aim of the clustering was to select the best sets of variables for prediction purposes, while reducing overfitting and the complexity of the model. For that purpose, a correlation matrix was generated from all the weather predictor summaries using Pearson correlation implemented in the cor function in base R. The correlation matrix was converted into a distance matrix which was then used for generating a hierarchical tree. Finally, clusters were generated by dividing the tree into 3 to 8 groups (k).

**Correlation approach**: In the correlation method, a coefficient of correlation was calculated for each weather variable and the response variable. As presence of bacteria is binary variable and weather factors are continuous variables, point biserial correlation (r_pbi_) was calculated as measure of correlation (Tate, 1954). For each lag period, the absolute values of the correlation between response and each member of the cluster of highly collinear variables were averaged to get a cluster mean. Then, cluster means were averaged across all clusters to get a single grand average of absolute correlation per lag period. The lag period with the highest resulting grand average was considered the best time‑lag.

**Regression approach**: In the regression approach, the response variable was first modeled using independent predictors that are not related to weather (and thus without time-lag). The residuals of the regression were then used as response variable in another regression with weather variables as independent predictors. As presence of bacteria is in binary scale and the residuals of binary regression are not meaningful for another binary regression, we used log of Colony Forming Units (lCFU) as response variable and modeled it using a gaussian distribution. For the first regression, lCFU was modeled with distance from point of inoculation and weeks post inoculation. The residuals of this model were used as the response variable with the linear combination of weather factors as predictors. A large number of regressions were performed for different sets of weather predictors, each with different combinations of one member of each cluster of highly collinear variables. All possible combinations of weather predictors were exhausted for each lag period. Bayesian information criterion (BIC) from all the regressions for each time‑lag was averaged and the lag period with the lowest average BIC was selected as the best time‑lag period.

Using both the correlation and regression method, the best time‑lag was obtained at 3 days period (Table S9-1, Figure S9-5). Subsequently, weather summaries representing 3 days lag was used for subsequent modeling.

Table

S9‑1. Average correlation of clustered weather variable to presence of bacteria. The highest grand average per cluster is underlined.

| **Lag in days** | **Number of clusters** | | | | | |
| --- | --- | --- | --- | --- | --- | --- |
|  | **3** | **4** | **5** | **6** | **7** | **8** |
| **0** | 0.147 | 0.185 | 0.140 | 0.120 | 0.157 | 0.170 |
| **1** | 0.127 | 0.148 | 0.126 | 0.100 | 0.129 | 0.145 |
| **2** | 0.123 | 0.160 | 0.120 | 0.117 | 0.133 | 0.144 |
| **3** | 0.150 | 0.188 | 0.204 | 0.170 | 0.167 | 0.181 |
| **4** | 0.146 | 0.113 | 0.122 | 0.145 | 0.126 | 0.123 |
| **5** | 0.143 | 0.103 | 0.066 | 0.095 | 0.084 | 0.093 |
| **6** | 0.143 | 0.085 | 0.086 | 0.100 | 0.097 | 0.100 |


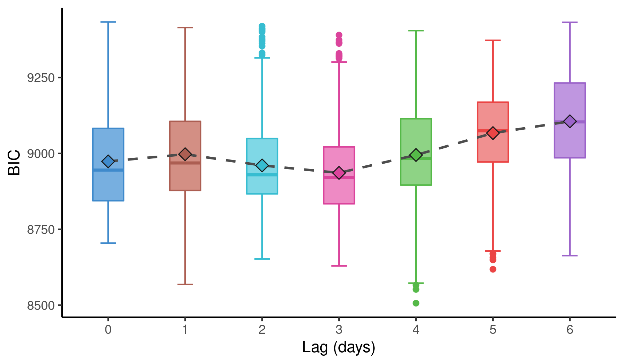


Figure

S9‑5. Boxplot of BIC by modeling residuals of CFU to distance and time to clustered weather parameters across 0 to 6 days lag period. Diamonds represent mean values.

# Significance test for fixed predictors

Table

S10‑2. Wald’s Chi squared values for the fixed predictors in the final model.

|  | **Df** | **Type III Chi-sq** | **Pr(>Chi-sq)** | **Type II Chi-sq** | **Pr(>Chi-sq)** |
| --- | --- | --- | --- | --- | --- |
| **(Intercept)** | 1 | 17.230 | <0.001 |  | <0.001 |
| **Time (week)** | 1 | 202.064 | <0.001 | 436.021 | <0.001 |
| **Distance (meter)** | 1 | 76.898 | <0.001 | 51.843 | <0.001 |
| **Gene_XopJ2+** | 1 | 30.430 | <0.001 | 230.091 | <0.001 |
| **Temp.mean** | 1 | 40.232 | <0.001 | 104.035 | <0.001 |
| **Humidity.mean** | 1 | 21.334 | <0.001 | 73.339 | <0.001 |
| **Rain.sum** | 1 | 29.669 | <0.001 | 29.669 | <0.001 |
| **Time:Gene_XopJ2+** | 1 | 49.415 | <0.001 | 49.415 | <0.001 |
| **Distance:Gene_XopJ2+** | 1 | 27.748 | <0.001 | 27.748 | <0.001 |
| **Gene_XopJ2+:Humidity.mean** | 1 | 15.302 | <0.001 | 15.302 | <0.001 |
| **Gene_XopJ2+:Temp.mean** | 1 | 13.091 | <0.001 | 13.091 | <0.001 |

Notations: Chi-sq: chi squared value; Df: Degrees of Freedom; Pr(>Chi-sq): p-value (marginal significance); Time: Weeks post inoculation; Distance: Distance from point of inoculation in meters; Gene: Gene-type of the bacteria (0 for absence of *xopJ2* and 1 for presence of *xopJ2* gene); Temp.mean: Average weekly temperature (standardized unit); Humidity.mean: Average weekly relative humidity; Rain.mean: Total weekly precipitation

# Selection of random covariates

To check the usefulness of a random covariate in a model, numbers of models were generated by stepwise reduction of random covariates from the full model. The significance of a covariate was tested by comparing the model to a step-down model without the covariate. For nested covariates, the simpler model consisted of all higher-level covariates. For top level covariate, the random effect consisted of a constant only.

Table S11-3 shows model selection parameters for full model and models with stepwise reduction of random covariates.

Table

S11‑3. Model selection parameters for selection of random predictors.

|  | **Null model** | | **Year** | | **Year (Dir)** | | **Rep (Year)** | | **ID (Rep * Year)** | | **Year, Rep (Year) and ID (Rep * Year)** | | **Year, Rep (Year), ID (Rep * Year) and Year (Dir)** | |  |
| --- | --- | --- | --- | --- | --- | --- | --- | --- | --- | --- | --- | --- | --- | --- | --- |
| **Number of parameters** | | 12 | | 12 | | 12 | | 12 | | 12 | | 14 | | 15 | |
| **AIC** | | 3013 | | 2844 | | 2785 | | 2807 | | 2441 | | 2323 | | 2315 | |
| **BIC** | | 3089 | | 2920 | | 2861 | | 2883 | | 2517 | | 2411 | | 2410 | |
| **Log likelihood** | | -1494 | | -1410 | | -1380 | | -1391 | | -1208 | | -1147 | | -1143 | |
| **Deviance** | | 2989 | | 2820 | | 2761 | | 2783 | | 2417 | | 2295 | | 2285 | |
| **LR Chi-sq** | | NA | | 169 | | 59 | | 37 | | 366 | | 122 | | 9 | |
| **DF (LR Chi-sq)** | | NA | | 0 | | 0 | | 0 | | 0 | | 2 | | 1 | |
| **Pr(> LR Chi-sq)** | | NA | | < 0.001 | | < 0.001 | | < 0.001 | | < 0.001 | | < 0.001 | | < 0.001 | |

Notations: Year: year of experiment; Dir: direction of growth; Rep: replication; ID: plant ID; AIC: akaike information criterion; BIC: bayesian information criterion; LR: likelihood ratio; Chi-sq: chi squared; DF: degrees of Freedom, Pr: probability.

Note that the model with “Rep (Year)” is compared to the model with “Year” and the rest are compared to the model with elements in the column immediately left to it in the table.

# Rate of dispersal

From logistic equation S2-, we have:


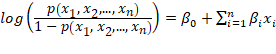
 eq

S12‑

where, μ is probability predicted by the model, are independent predictors of the model including interactions, are respective estimates, and β_0_ is coefficient of the intercept. Bacteria are assumed to be present when probability exceeds a certain constant cut-off threshold probability, e.g., . Replacing probability (p) with a constant, the entire left-hand side (LHS) becomes a constant. So, equation S12- becomes,


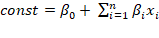
 eq

S12‑

Next, we can expand the right-hand side (RHS) of the equation. Let x^t^ be predictors which have significant interaction with weeks post inoculation (time) and x^d^ be predictors which have significant interaction with distance from point of inoculation (dist). If we assume constant weather condition, fixed direction of spread and a specific gene-type (gene), then all β_i_x_i_ terms except those containing time and dist will be constant. If we assume a constant dispersal velocity, then we can also remove the dist:time interaction. In such a case, for variable date and time, equation S12- can be expanded as,


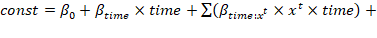


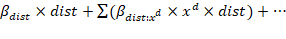
 eq

S12‑

where, ⋯ includes all other terms of the equation and is a constant under previously stated assumptions. Simplifying equation S12-, we get,

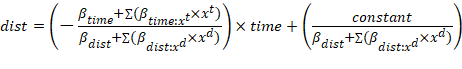
 eq

S12‑

which is in the form , where the slope (m) represents distance changed per unit time, and hence is the dispersal velocity (V) of the bacteria. The rate can also be calculated by taking derivative of dist with respect to time, which gives,


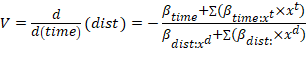
 eq

S12‑

Equation S12- also shows that factors that have significant interactions with distance and time alter the dispersal velocity of pathogen. Next, we can calculate the ratio of the dispersal velocity with and without presence of a gene by taking ratio of the slopes. If only gene has significant interaction with date and time,


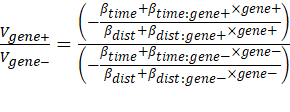
 eq

S12‑

In the logistic regression, if gene– is the base level and gene+ is only other level of gene,


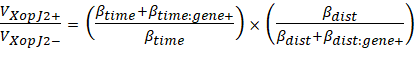
 eq

S12‑

# Average levels of weather predictors in full model


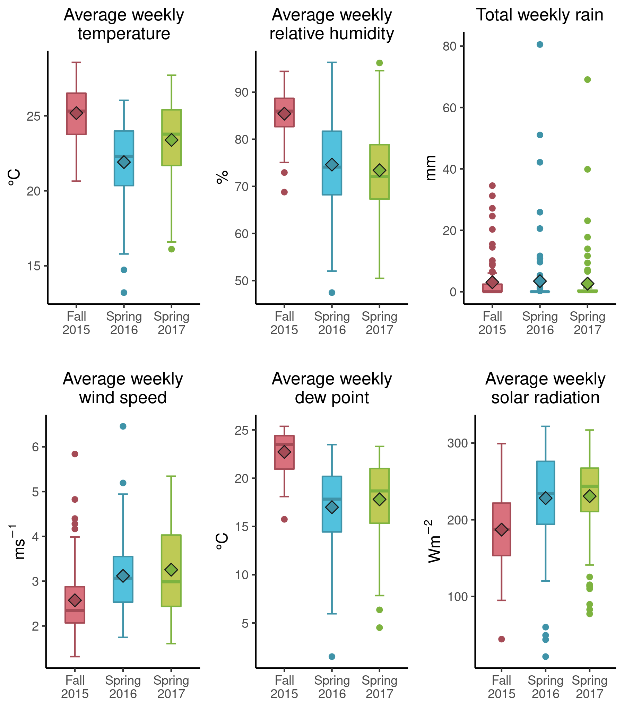


Figure

S14‑6. Ranges of weather experienced during the experimental period. Diamonds represent the mean values.

Table

S14‑4. Scaling parameters for weather variables used in the model.

|  | **Mean** | **Standard deviation** |
| --- | --- | --- |
| **Average weekly temperature (°C)** | 23.50 | 2.77 |
| **Average weekly wind speed (ms^-1^)** | 2.98 | 0.91 |
| **Average weekly relative humidity (%)** | 77.85 | 9.72 |
| **Average weekly dew point (°C)** | 19.19 | 4.40 |
| **Average weekly solar radiation (Wm^-2^)** | 215.23 | 59.63 |
| **Total weekly rain (mm)** | 3.07 | 9.48 |

# Recovery of XopJ2–


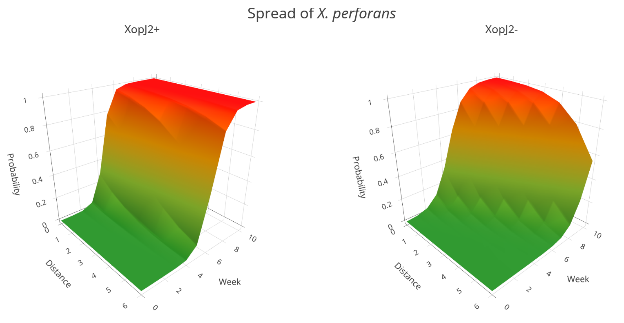


Figure

S13‑7. 3d surface plot showing the probability of presence of *Xp* over time and distance as predicted by the model. Note that although XopJ2+ is already established at larger distance earlier than XopJ2–, the presence of XopJ2– is increasing nonetheless.

# Effective population and detection threshold

The simulation of gene frequency needs three input parameters: relative fitness (w_1_ and w_2_), effective population size (N_e_) and initial frequency of gene (f_0_). The value of w_1_ and w_2_ were obtained from the final model.

N_e_ in this paper is defined in Wright-Fisher sense, but for haploid population. In simple terms, N_e_ is the constant size of bacterial population that contributes to next generation of bacteria in a field. While there is consensus on what an N_e_ on the field would be for *Xp*, we set it at 1×10^8^ in our simulation for two reasons: (i) it is close to N_e_ estimate reported for plant pathogens, albeit in a different context, by Bobay and Ochman (2018), and (ii) setting a number of a higher order was not possible due to extremely large size of matrices needed for computational sampling process. Moreover, N_e_ affects sampling causing random drift, but if does not directly affect change in gene frequency due to selection.


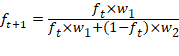
 eq

S12‑

where, f is frequency of gene,

t is number of generations, and

w_1_ and w_2_ are relative fitness with and without genes.

So, the accuracy and preciseness of the value of N_e_, when sufficiently large to mitigate effects of random drift, is not important for this simulation based on selection.

The f_0_ of a gene is the fraction of the bacterial population in which the gene is present at generation 0 at the beginning of the simulation. For a horizontally transferred gene, f_0_ would be extremely low, as it is a relatively rare event. It is helpful to use the detection threshold (f_d_) for a gene as its f_0_. f_d_ is the lowest minimum gene frequency at which its presence can be identified. f_d_ is the property of sampling, isolation, and identification techniques, and does not change as the population size is changed (e.g., from a single field to a county/state). For the field condition, we set the f_d_ for *xopJ2* at 10^‑7^, meaning we first identify the bacterial strain with a novel gene once its abundance in the field exceeds one in ten million. It is difficult to estimate the real f_d_ for *xopJ2* in *Xp* in the field and we used 10^‑7^ as we were constrained by the value of N_e_ for sampling. f_0_ affects the number of generations required for fixation the positively selected gene, but it does not affect the rate of increase in gene frequency. As f_0_ is changed in log scale, the number of years required for the fixation of a gene changes in linear scale.

## Supplementary References

Akaike, H. (1973) Information theory and an extension of the maximum likelihood principle. In Petrov, B. and Csaki, F. (eds). Budapest: Akademiai Kiado, pp. 267–281.

Allouche, O., Tsoar, A., and Kadmon, R. (2006) Assessing the accuracy of species distribution models: prevalence, kappa and the true skill statistic (TSS). *J Appl Ecol* **43**: 1223–1232.

Baayen, R.H., Davidson, D.J., and Bates, D.M. (2008) Mixed-effects modeling with crossed random effects for subjects and items. *J Mem Lang* **59**: 390–412.

Bobay, L.-M. and Ochman, H. (2018) Factors driving effective population size and pan-genome evolution in bacteria. *BMC Evol Biol* **18**: 153.

Bolker, B.M., Brooks, M.E., Clark, C.J., Geange, S.W., Poulsen, J.R., Stevens, M.H.H., and White, J.-S.S. (2009) Generalized linear mixed models: a practical guide for ecology and evolution. *Trends Ecol Evol* **24**: 127–135.

Cohen, J. (1960) A Coefficient of Agreement for Nominal Scales: *Educ Psychol Meas* **20**: 37–46.

Denuit, M., Mesfioui, M., and Trufin, J. (2019) Bounds on Concordance-Based Validation Statistics in Regression Models for Binary Responses. *Methodol Comput Appl Probab* **21**: 491–509.

Funk, S., Camacho, A., Kucharski, A.J., Lowe, R., Eggo, R.M., and Edmunds, W.J. (2019) Assessing the performance of real-time epidemic forecasts: A case study of Ebola in the Western Area region of Sierra Leone, 2014-15. *PLoS Comput Biol* **15**: e1006785.

Goodman, L.A. and Kruskal, W.H. (1954) Measures of Association for Cross Classifications. *J Am Stat Assoc* **49**: 732–764.

Kendall, M.G. (1938) A New Measure of Rank Correlation. *Biometrika* **30**: 81–93.

Kraemer, H.C. (2006) Correlation coefficients in medical research: from product moment correlation to the odds ratio. *Stat Methods Med Res* **15**: 525–545.

Leclerc, M., Doré, T., Gilligan, C.A., Lucas, P., and Filipe, J.A.N. (2014) Estimating the Delay between Host Infection and Disease (Incubation Period) and Assessing Its Significance to the Epidemiology of Plant Diseases. *PLoS One* **9**: e86568.

Liu, C., White, M., and Newell, G. (2011) Measuring and comparing the accuracy of species distribution models with presence–absence data. *Ecography* **34**: 232–243.

Matthews, B.W. (1975) Comparison of the predicted and observed secondary structure of T4 phage lysozyme. *Biochim Biophys Acta* **405**: 442–451.

Molenberghs, G. and Verbeke, G. (2007) Likelihood Ratio, Score, and Wald Tests in a Constrained Parameter Space. *The American Statistician* **61**: 22–27.

Nakagawa, S., Johnson, P.C.D., and Schielzeth, H. (2017) The coefficient of determination R^2^ and intra-class correlation coefficient from generalized linear mixed-effects models revisited and expanded. *J R Soc Interface* **14**: 20170213.

Peirce, C.S. (1884) The numerical measure of the success of predictions. *Science* **ns-4**: 453–454.

Piepho, H.-P. (2019) A coefficient of determination (R2) for generalized linear mixed models. *Biometrical Journal* **61**: 860–872.

Pinheiro, J.C. and Bates, D.M. (2000) Theory and computational methods for linear mixed-effects models. In *Mixed-effects models in S and S-PLUS*. New York: Springer, pp. 57–96.

Schwarz, G. (1978) Estimating the Dimension of a Model. *Ann Stat* **6**: 461–464.

Somers, R.H. (1962) A New Asymmetric Measure of Association for Ordinal Variables. *Am Sociol Rev* **27**: 799–811.

Stuart, A. (1953) The Estimation and Comparison of Strengths of Association in Contingency Tables. *Biometrika* **40**: 105–110.

Tate, R.F. (1954) Correlation Between a Discrete and a Continuous Variable. Point-Biserial Correlation. *Ann Math Stat* **25**: 603–607.

Wald, A. (1943) Tests of Statistical Hypotheses Concerning Several Parameters When the Number of Observations is Large. *Trans Am Math Soc* **54**: 426–482.

Wearing, H.J., Rohani, P., and Keeling, M.J. (2005) Appropriate Models for the Management of Infectious Diseases. *PLoS Med* **2**: e174.

Youden, W.J. (1950) Index for rating diagnostic tests. *Cancer* **3**: 32–35.
